# Metabolic Homeostasis of Amino Acids and Diabetic Kidney Disease

**DOI:** 10.3390/nu15010184

**Published:** 2022-12-30

**Authors:** Luokun Liu, Jingge Xu, Zhiyu Zhang, Dongwen Ren, Yuzheng Wu, Dan Wang, Yi Zhang, Shuwu Zhao, Qian Chen, Tao Wang

**Affiliations:** 1 State Key Laboratory of Component Based Chinese Medicine, Tianjin University of Traditional Chinese Medicine, 10 Poyanghu Road, Jinghai District, Tianjin 301617, China; 2 Haihe Laboratory of Modern Traditional Chinese Medicine, Tianjin University of Traditional Chinese Medicine, 10 Poyanghu Road, Jinghai District, Tianjin 301617, China; 3 School of Intergrative Medicine, Tianjin University of Traditional Chinese Medicine, 10 Poyanghu Road, Jinghai District, Tianjin 301617, China

**Keywords:** amino acids, metabolic homeostasis, metabolites, diabetic kidney disease, intestinal microecology

## Abstract

Diabetic kidney disease (DKD) occurs in 25–40% of patients with diabetes. Individuals with DKD are at a significant risk of progression to end-stage kidney disease morbidity and mortality. At present, although renal function-decline can be retarded by intensive glucose lowering and strict blood pressure control, these current treatments have shown no beneficial impact on preventing progression to kidney failure. Recently, in addition to control of blood sugar and pressure, a dietary approach has been recommended for management of DKD. Amino acids (AAs) are both biomarkers and causal factors of DKD progression. AA homeostasis contributes to renal hemodynamic response and glomerular hyperfiltration alteration in diabetic patients. This review discusses the links between progressive kidney dysfunction and the metabolic homeostasis of histidine, tryptophan, methionine, glutamine, tyrosine, and branched-chain AAs. In addition, we emphasize the regulation effects of special metabolites on DKD progression, with a focus on causality and potential mechanisms. This paper may offer an optimized protein diet strategy with concomitant management of AA homeostasis to reduce the risks of DKD in a setting of hyperglycemia.

## 1. Introduction

Diabetic kidney disease (DKD) has been recognized as a predominantly serious diabetic microvascular complication, affecting almost 40% of people with diabetes [1,2]. DKD is the key risk factor for end-stage kidney disease (ESKD) and diabetic mortality. Recently, as the prevalence of DKD grows continuously, the number of patients with ESKD caused by DKD is increasing, with an irreversible endpoint, and of note is that the 3-year and 5-year renal survival rates were 74.5% and 22.6%, respectively, in a small-scale clinical prognostic evaluation study in China [3]. Currently, clinical therapy and nutrition strategy mainly focus on blood glucose control, blood pressure control, lipid control, and diet management [4]. Within these, dietary management plays a critical role in retarding renal-function decline and prevention of DKD progression to ESKD [5].

Amino acids (AAs), as the fundamental components of protein, are important signaling molecules in modulation of energy and metabolic homeostasis [6]. There is a growing body of evidence of kidney function on AA metabolic homeostasis, including AA synthesis, degradation, filtration, reabsorption, and urinary excretion [7]. Disruption of AA homeostasis conversely affects kidney-dominated AA metabolism. In response to this disruption, disorder of the relative cellular signaling pathway network directly or indirectly drives development of DKD [8]. On one hand, disorder of functional AA levels in plasma reflects renal dysfunction in diabetes patients; on the other hand, disturbance in AA metabolic homeostasis causes abnormal accumulation of harmful metabolites or activation of metabolism enzymes, which may trigger cellular signaling in DKD progressions such as oxidative stress, inflammation, fibrosis, and apoptosis. Thus, it is essential to maintain circulating AAs and their metabolites at an appropriate level to ensure renal function under hyperglycemia.

The capability of sensing and responding to changes in AAs is mediated through a complex regulatory network in the body. Abundance or deficiency of AAs is always detected with various sensors via the mechanistic target of the rapamycin complex 1 (mTORC1), general control nonderepressible 2 (GCN2), or fibroblast growth factor 21 (FGF21) signaling pathway [9]. For instance, some AAs, such as leucine and arginine, are directly sensed [10], whereas others, such as methionine, are sensed in the form of their metabolites [11]. Therefore, a strict low-protein diet of only AAs may induce several nutritional risks that result in functional AA deficiency, rather than benefits for renal protection in DKD patients [12]. In contrast, excess protein intake may not only aggravate renal metabolic burden but also increase the risk of progressive renal dysfunction activated via accumulated uremic toxins in body fluids. As is well-known, gut microbial diversity is decreased with a remarkably different composition profile and an impaired intestinal barrier in diabetes patients, contributing to a harmful uremic circumstance [13,14]. Thus, uremic toxins have emerged as a key factor to explain DKD. AA metabolism is one of the most important sources of uremic toxins such as indoxyl sulfate (IS), phenyl sulfate (PS), and imidazole propionate (IMP) [15,16].

Despite increasing awareness of the contribution of AA metabolic homeostasis and the metabolites of these AAs to DKD progression, mechanistic links still lack sufficient evidence. In this review, we: (1) Discuss the links between progressive kidney dysfunction and AA metabolic homeostasis; (2) Verify the regulation effects of special metabolites on DKD progression with a focus on causality and potential mechanisms; (3) Summarize potential biological markers from AA metabolism to meet the urgent demands of poor DKD prognosis; (4) Expect prospects for an optimized protein-diet therapy.

## 2. Histidine and Microbially Produced Imidazole Propionate in the Gut

**Figure 1:**
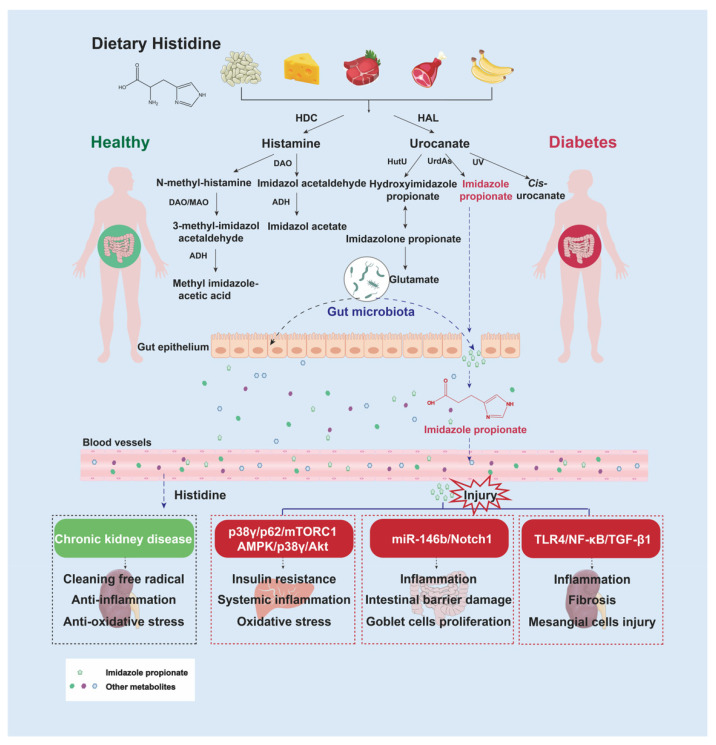
Gut microbiota regulation of histidine metabolism in health and disease. In the healthy state, histidine is mainly metabolized into methyl imidazole acetic acid, imidazole acetate, glutamate, and cis-urocanate, whereas in the diabetic state, due to dysbiosis of gut microbiota, IMP is produced from histidine by diabetes-associated bacteria. Normally, histidine has a wide range of physiological benefits, such as hydroxyl radical and singlet oxygen scavenging and anti-inflammation and antioxidative stress protection in CKD patients. However, under colitis conditions, IMP has been confirmed to induce intestinal inflammation and impair the intestinal barrier through inhibition of the miR-146b/Notch1 axis. Under type 2 diabetes conditions, IMP was demonstrated to impair glucose tolerance through suppression of the insulin-signaling pathway and stimulation of the inflammation and oxidative stress pathways. Additionally, IMP is further associated with DKD progression through activation of the TLR4 pathway. Abbreviations: ADH, alcohol dehydrogenase; DAO, diamine oxidase; HAL, histidine ammonia-lyase; HDC, histidine decarboxylase; HutU, urocanase gene; MAO, monoamine oxidase; NF-κB, nuclear factor-κB; TGF-β1, transforming growth factor β1; UrdAs, urocanate reductases; UV, ultraviolet.

Histidine is a conditional essential AA for humans, and diet sources rich in histidine include beans, cheese, beef, chicken, and bananas. After absorption, the major pathway of histidine catabolism starts with decarboxylation and deamination via histidine decarboxylase and histidine ammonialyase, leading to production of methyl imidazole acetic acid, imidazole acetate, glutamate, IMP, and cis-urocanate [17,18]. Bioactivities of histidine are customarily reported as free radical scavenging and prevention of LDL cholesterol oxidation [19]. A low-protein diet is recommended for DKD patients in a clinical setting. However, histidine is suggested as an essential AA for patients with chronic kidney disease (CKD). Histidine supplementation appears to be beneficial to prevent protein–energy waste, inflammation, and oxidative stress in CKD subjects [20], whereas histidine-derived IMP that is metabolized by gut microbiota is clinically recognized as an indicator of inflammatory bowel disease and metabolic syndrome [21]. IMP is confirmed to induce intestinal inflammation and damage the intestinal barrier through the miR-146b/Notch 1 pathway in high-histidine-fed mice [22]. Under diabetic circumstances, IMP is observed to induce insulin resistance and participate in the diabetic pathological process (Figure 1).

A targeted metabolite analysis showed higher plasma concentrations of IMP in type 2 diabetes patients and genetically diabetic mice [23]. A further mechanistic study subsequently elucidated that IMP impaired insulin-receptor substrate-mediated insulin signaling through activation of the p38γ/p62/mTORC1-dependent pathway [23] and suppressed metformin-induced AMPK phosphorylation via activation of the p38γ/Akt-dependent pathway [24] (Figure 1). Most notably, it is the altered microbial metabolism of histidine, rather than histidine intake, that contributes to type 2 diabetes via generation of IMP. Consequently, increased serum IMP levels have been featured among subjects with the Bacteroides 2 enterotype [25]. This human enterotype has been linked to low microbiome gene richness and proinflammatory microbiota composition, which also occurs in DKD mice [26]. Altogether, DKD patients suffer from long-term chronic inflammation, partially owing to diabetic dysbiosis of gut microbiota. From the aspect of disordered histidine metabolism, low-grade inflammation in development of diabetic progressive kidney dysfunction is likely to be associated with increased circulating IMP levels. In the latest study, IMP was verified to have harmful effects on mesangial cells that aggravate DKD progression through activation of toll-like receptor 4 (TLR4) in db/db mice [27]. Thus, it is necessary for potential DKD patients to sustain gut microbiota balance and protect the intestinal barrier in advance, achieving the goal of reducing harmful circulating metabolites, such as IMP, from intestinal leakage. Importantly, histidine intake should be ensured to be satisfied with the function demand of DKD patients. However, clinic histidine metabolomics of diabetic and DKD patients with or without gut microbial modulation need to be evaluated further.

## 3. Tryptophan and Microbially Produced Indoles Sulfate in the Gut

**Figure 2:**
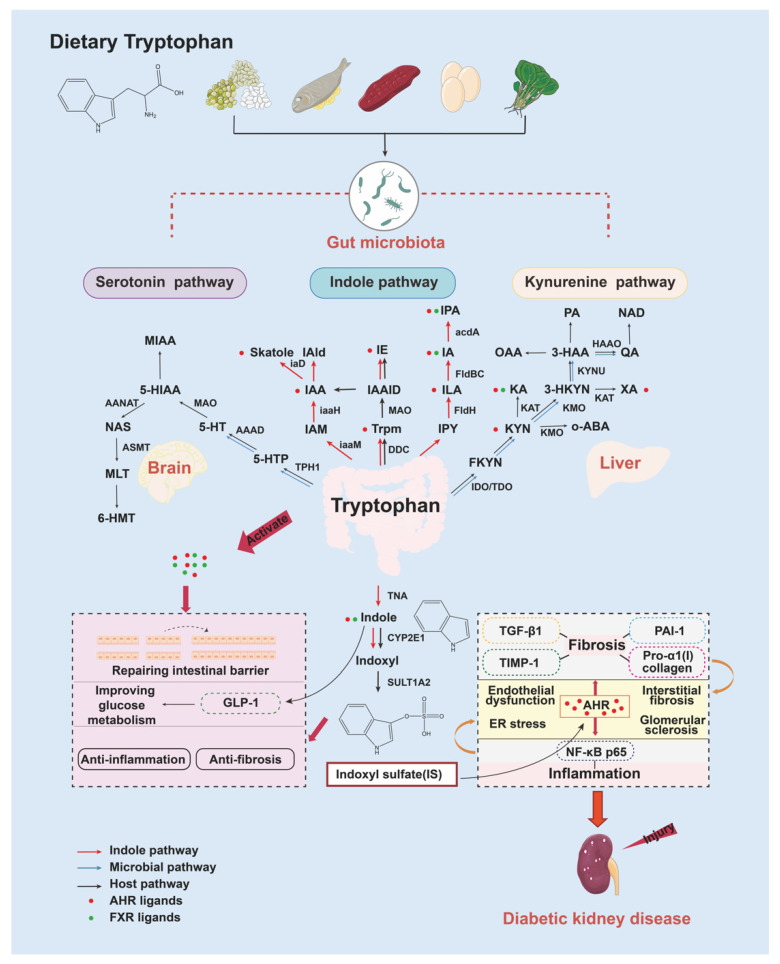
Gut microbiota regulation of tryptophan metabolism in health and disease. There are three main metabolic pathways for tryptophan. Serotonin pathway: tryptophan is catalyzed with tryptophan hydroxylase1 (TPH1) in the enterochromaffin cell to produce peripheral 5-HT, which is involved in regulation of gastrointestinal functions and circadian rhythms. Indole pathway: tryptophan is directly metabolized to indole and indole derivatives via intestinal flora. Indole derivatives include IAA, ILA, 3-indoleacrylate acid (IA), indole-3-carboxaldehyde (IAld), tryptamine (Trpm), IS, etc. Indole-pathway metabolites play an important role in regulation of intestinal immunity and metabolic homeostasis. Particularly, IS activates AHR and contributes to progression of renal-function decline. Kynurenine pathway: more than 95% of tryptophan is metabolized via this pathway. Tryptophan is metabolized by three rate-limiting enzymes, IDO1, IDO2, and tryptophan-2,3-dioxygenase (TDO), to produce formyl-kynurenine (FKYN), which is then catalyzed by formylase to produce KYN and the downstream metabolites kynurenic acid (KA), picolinic acid (PA), nicotinamide adenine dinucleotide (NAD), and xanthurenic acid (XA). Abbreviations: 3-HAA, 3-hydroxyanthranilate; 3-HKYN, 3-hydroxykynurenine; 5-HIAA, 5-hydroxyindole acetic acid; 5-HTTP, 5-hydroxytryptophan; 6-HMT, 6-chloromelatonin; AAAD, aromatic amino-acid decarboxylase; AANAT, *N*-acetyltransferase; acdA, acyl-CoA dehydrogenase; ASMT, acetylserotonin-O-methyltransferase; DDC, dopa decarboxylase; FldBC, phenyllactate dehydratase; FldH, phenyllactate dehydrogenase; FXR, farnesoid X receptor; HAAO, 3-hydroxyanthranilate 3,4-dioxygenase; iaaH, indole-3-acetic acid hydrazide; IAAID, indole-3-acetaldehyde; iaaM, tryptophan monooxygenase gene; iaD, indoleacetic acid oxidase; IAM, indole-3-acetamide; IE, indole-3-ethanol; IPY, indolepyruvate; KAT, kynurenic aminotransferase; KMO, kynurenine-3-monooxygenase; KYNU, kynureninase; MAO, monoamine oxidase; MIAA, 5-methoxyindole acetate; MLT, melatonin; NAS, *N*-acetylserotonin; OAA, 2-oxoadipic acid; o-ABA, o-aminobenzoic acid; QA, quinolinate; Skatole, 3-methylindole; TnaA, tryptophanase.

Tryptophan is a fundamental aromatic AA that is abundant in beans, fish, beef, eggs, and spinach. Tryptophan metabolism follows three major pathways under direct or indirect control of microbiota: (1) the serotonin (5-hydroxytryptamine, 5-HT) pathway, which produces serotonin derivatives in enterochromaffin cells via tryptophan hydroxylase; (2) the indole pathway, which transfers tryptophan to indole series derivatives via the microbial community; and (3) the kynurenine (KYN) pathway, which produces kynurenine derivatives in immune and epithelial cells via indoleamine 2,3-dioxygenase (IDO) [28,29,30]. Among them, AAs such as tryptamine, indole, and indoleacetic acid (IAA) can enhance epithelial barrier functions via decreased intestinal permeability, possibly mediated by the pregnane X receptor in vitro [31]; similarly, indole can improve the glucose metabolism of patients with metabolic syndrome through modulation of glucagon-like peptide-1 (GLP-1) secretion. Indole, indole-3-lactic acid (ILA), and indole-3-propionic acid (IPA) have clinical antioxidative and anti-inflammatory effects [32,33], whereas IS, which is produced in the liver from indole via the actions of cytochrome P450 2E1 (CYP2E1) and sulfotransferase 1A2 (SULT1A2), has cytotoxic effects in high concentrations (Figure 2).

Levels of IS have been reported to increase in the early stages of CKD in patients, and they rise relentlessly with CKD progression [34]. In addition, the levels of IS in the ischemic acute renal injuries of rats increased by four times compared with those of sham rats [35]. As a typical aryl hydrocarbon receptor (AHR) ligand, IS activates AHR and then induces endothelial dysfunction, endoplasmic reticulum (ER) stress, podocyte injury, interstitial fibrosis, and glomerular damage in experimental animal models of metabolic syndrome [36]. In progression of renal-function decline in CKD mammal models, IS not only stimulates nuclear translocation of phosphorylated NF-κB p65 to induce activation of NF-κB signaling [37] but also aggravates renal fibrosis via upregulation of TGF-β1, plasminogen activator inhibitor-1 (PAI-1), the tissue inhibitor of matrix metalloproteinase-1 (TIMP-1), and pro-α1 (I) collagen [38,39] (Figure 2). Due to IS production dependence on indole-producing bacteria (*Bacteroides* spp. and *Clostridium* spp.) in the gut, IS was undetectable in germ-free mice [33]. As a consequence, manipulation of gut-microbial tryptophan catabolism may be a potential strategy to lower circulating levels of IS. However, whether elimination of IS from tryptophan catabolism will be beneficial in renal diseases needs to be examined in future clinical studies.

## 4. Methionine and Its Metabolite, Homocysteine

**Figure 3:**
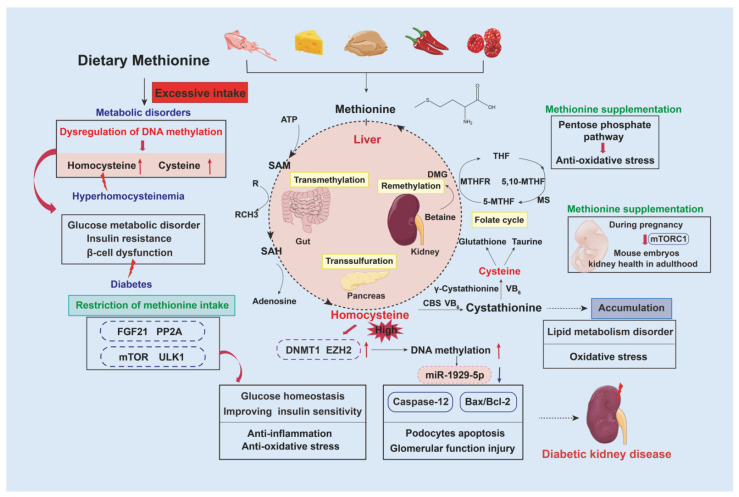
Metabolic regulation of methionine supplementation and restriction. Methionine is catabolized by adenosine triphosphate (ATP) to SAM, which serves as a universal donor for methyl transfer reactions. SAH is produced as a methyl-transfer-reaction product that utilizes SAM as a methyl donor. Homocysteine is formed from reversible hydrolysis of SAH. Levels of homocysteine are regulated through remethylation of homocysteine to methionine via the methionine synthase (MS) enzyme and trans-sulfuration of homocysteine to cystathionine via the cystathionine β-synthase (CBS) enzyme. Abbreviations: 5-MTHF, 5-methyltetrahydrofolate; 5,10-MTHF, 5,10-methylenetetrahydrofolate; DMG, 1,2-dimyristoyl-sn-glycerol; MTHFR, 5,10-methylenetetrahydrofolate reductase; THF, tetrahydrofolate.

Methionine is an essential sulfur-containing AA enriched in squid, cheese, chicken breast, pepper, and raspberry. Methionine is catalyzed into S-adenosylmethionine (SAM), S-adenosylhomocysteine (SAH), homocysteine (Hcy), and cysteine (Cys), which occur primarily in the liver, kidney, small intestine, and pancreas [40]. Most of the enzymes in this metabolic reaction are hepatic-specific, including methionine adenosyltransferase, S-adenosylhomocysteinase, cystathionine β-synthase, and cystathionine γ-lyase, which belong to the methionine and folate cycles and the trans-sulfuration pathway [41]. As reported previously, methionine, which acts as a reactive oxygen-species scavenger, is implicated in caloric restriction and aging [42]. Consistent evidence has shown that methionine supplementation attenuates oxidative stress resistance, possibly through the pentose phosphate pathway in vitro [43]. Additionally, methionine supplementation during pregnancy reverses the negative effect of maternal malnutrition on the developing kidney, contributing to nephrogenesis and later kidney health in adulthood; this was mediated through the mTORC1 pathway in a calorie-restricted-diet mouse model [44]. Despite consumption of methionine being essential to sustain life, basic experimental evidence has shown that methionine restriction provides benefits in impairment of glucose disorder, increasing insulin sensitivity, amelioration of oxidative stress and inflammation, and extension of healthy lifespan [45,46]. Subsequent studies have also pointed out that the effects of limiting methionine in the diet of rats depend on stimulation of FGF21, protein phosphatase 2A (PP2A), and autophagy-related mTOR and UNC-51-like kinase 1 (ULK1) [11] (Figure 3).

SAM is a methyl donor for DNA or protein methylation. Excessive intake of methionine destroys its original metabolic homeostasis, leading to abnormal accumulation of Cys and Hcy, as well as aberrant DNA methylation. It has been reported that beneficial effects of methionine restriction in mammals were reversed with Cys supplementation. Abnormal DNA methylation is closely associated with glucose metabolism, insulin resistance, and β-cell dysfunction, contributing to occurrence and development of diabetes [47]. In addition, high methionine intake may induce hyperhomocysteinemia (HHcy), a risk factor for cardiovascular and neurological disorders. HHcy has also been reported to be associated with acceleration of CKD progression. The relevant study demonstrated that Hcy promotes podocytes apoptosis through regulation of the epigenetic modifiers DNA methyltransferase 1 (DNMT1) and enhancer of zeste homolog 2 (EZH2), which are recruited by c-Myc to miR-1929-5p promoter and silence miR-1929-5p expression [48] (Figure 3). Hcy also had a role in the development of acute kidney injury in *cbs^+/−^* mice. Hcy-overloaded mice developed more severe renal injuries after cisplatin injection and ischemia-reperfusion injury and showed more severe renal tubular cell apoptosis and decreased tubular cell proliferation [49]. Although there has been little specific study about the effects of methionine on DKD progression, extensive basic animal evidence confirmed that a high level of Hcy will injure both glomerules and tubules. Thus, we can speculate that high intake of methionine will accelerate renal progressive dysfunction of diabetes. To sum up, intensive methionine restriction is not suitable to maintain the life spans of various animal species; it is recommended that adequate content of methionine in food range from 0.17% to 0.25% [50,51]. Diets deficient in methionine are probably a beneficial nutritional strategy in patients with diabetes or DKD.

## 5. Glutamine

**Figure 4:**
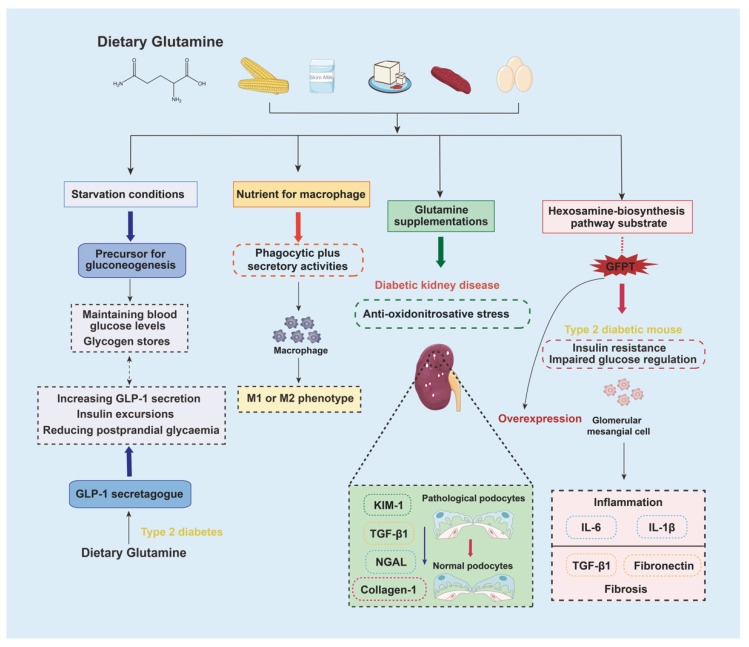
Metabolism and immune function of glutamine supplementation. In starvation or fasting stages, the liver plays the role of a major producer to a major glutamine consumer in order to maintain gluconeogenesis, and the whole body relies on the skeletal muscle’s stores to maintain glutamine levels. However, in diabetic stages, glutamine is helpful to accelerate GLP-1 secretion and reduce postprandial glycaemia. On the other hand, immune-system cells are extremely dependent on glutamine as an essential nutrient, especially during the macrophage activation process. Synthesis and secretion of proinflammatory cytokines by macrophages are also regulated based on glutamine availability, resulting in alteration of M1/M2 polarization. As a precursor of glutathione, glutamine is also useful to modulate oxidonitrosative stress conditions with decreased KIM-1, NGAL, TGF-β1, and collagen-1 production and protect renal function. Due to a crucial role in the hexosamine biosynthetic pathway, glutamine seems associated with overexpression of GFPT, which impairs glucose regulation and induces glomerular mesangial injury.

Glutamine is one of the most abundant and versatile AAs in human life, broadly existing in such foods as corn, milk, tofu, beef, and eggs. Normally, glutamine is a fundamental precursor to glutathione, which is a nonenzymatic antioxidant. Under fasting and starvation conditions, glutamine serves gluconeogenesis, helping the liver to maintain blood glucose levels following glycogen-store shortages [52]. As for type 2 diabetes patients, daily oral supplementation of glutamine decreased glycemia through increased GLP-1 secretion [53]. Glutamine is also an essential nutrient for macrophage activation [54]. Furthermore, glutamine supplementations presented protective effects against STZ-induced renal injury in rats through inhibition of oxidonitrosative stress as well as downregulation of the kidney injury molecule-1 (KIM-1), neutrophil gelatinase-associated lipocalin (NGAL), TGF-β1, and collagen-1 mRNA expressions [55] (Figure 4).

Although the beneficial activities of glutamine supplementation are already found in oxidative stress, immune function, and glucose homeostasis, answers to unignorable questions and evidence in vivo outcomes remain unclear [56,57,58]. In renal histology, it has been reported that diabetic rats supplemented with glutamine showed increases in proinflammatory interleukin (IL)-1β and IL-6 content in renal cortex and glomerulosclerosis alteration [59]. These adverse effects of glutamine may be expressed through its role of an important substrate in the hexosamine biosynthetic pathway, which is probably associated with glutamine: fructose-6-phosphate amidotransferase (GFPT) [60]. GFPT, the first and a rate-limiting enzyme in the hexosamine biosynthetic pathway, transfers the amide group from glutamine to fructose-6-phosphate to form glucosamine-6-phosphate. A body of studies have shown that overexpression of GFPT in diabetic mammals leads to hyperlipidemia, obesity, impaired glucose tolerance, and insulin resistance [61]. Additionally, GFPT has an impact on diabetic glomerular injury, which can be verified through upregulation of fibrotic TGF-β1 and fibronectin [62], as well as proinflammatory IL-1β and IL-6 in mesangial cells with GFPT overexpression in vitro (Figure 4). Collectively, excessive intake of glutamine may result in GFPT overexpression; thus, high glutamine load directly or indirectly activates the initial steps of DKD. Obviously, when glutamine is lacked, it is necessary to increase awareness regarding the risks associated with excessive dietary glutamine supplementation, especially among those who are diabetic or have transiently higher blood glucose levels.

## 6. Tyrosine and Microbially Produced Phenyl Sulfate in the Gut

**Figure 5:**
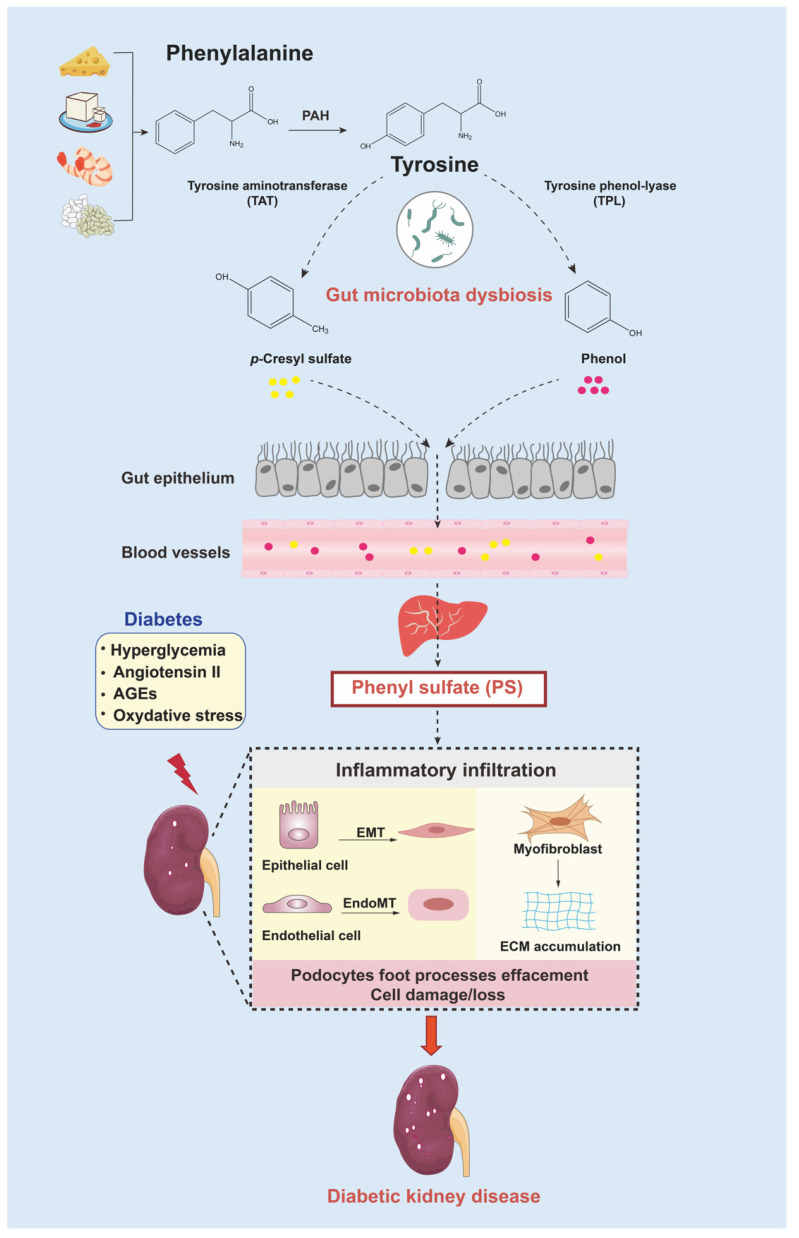
Microbially produced PS in the gut contributes to renal dysfunction in DKD. Generated tyrosine-derived toxins, such as PCS, and phenol easily directly cross the intestinal barrier, which is induced to be more permeable due to the effects of the deranged gut microbiome. PS is generated in the liver from phenol, which accumulates in plasma and has adverse effects on the vasculature and kidneys. In DKD, PS elicits inflammatory infiltration, accelerates glomerular-basement-membrane thickening, damages podocytes, and induces progression of renal failure. Abbreviations: EMT, epithelial-to-mesenchymal transition; EndoMT, endothelial-to-mesenchymal transition; PAH, phenylalanine hydroxylase.

Tyrosine is a semiessential AA that can be derived from either hydrolysis of dietary or tissue protein or hydroxylation of phenylalanine. Dietary phenylalanine usually exists in cheese, tofu, shrimp, and beans. Generally, tyrosine can be incorporated into proteins or degraded into fumarate and acetoacetate. The first step of the catabolic pathway is determined by the enzyme tyrosine aminotransferase (TAT) [63]. Then, gut flora participate in the subsequent fermentation and decomposition of tyrosine, resulting in production of uremic toxins, including *p*-cresyl sulfate (PCS) and phenol [64]. The two toxins are then metabolized into PS by the liver-specific enzyme (Figure 5). Studies have shown that disorders of tyrosine catabolism are associated with metabolic diseases. Subsequent research confirmed the contribution of tyrosine metabolism to type 2 diabetes progression. It has been demonstrated that elevation of serum tyrosine can inhibit the insulin pathway, which would then lead to insulin resistance [65,66]. Clinical findings further verified a highly positive correlation between plasma tyrosine levels (higher than 46 µmol/L) and type 2 diabetes morbidity rates, especially taking levels of high-density lipoprotein cholesterol and total cholesterol into consideration [67].

Recent metabonomic analysis showed that urine tyrosine concentration was significantly declined in type 2 diabetes patients with micro- to massive albuminuria [68]. In addition, some uric metabolites catabolized by tyrosine were altered in DKD subjects, such as through a decrease in homovanillic acid and an increase in hydroxyphenylacetic acid [69]. As for patients with chronic renal failure, plasma tyrosine levels were reduced, combined with increased phenylalanine levels [70]. A succession of findings illustrated that tyrosine and its metabolic homeostasis are relative to initiation and development of diabetic renal injury. Particularly, microbially produced intestinal PS is a promising predictor for incipient albuminuria in DKD patients. Further mechanism studies indicated that PS contributes to DKD renal failure through stimulation of inflammation, extracellular matrix (ECM) accumulation, and podocyte damage in db/db mice [71] (Figure 5). Collectively, PS is not only an early diagnosis biomarker but also a modifiable factor and therefore a potential target for DKD therapy.

## 7. Branched-Chain Amino Acids

**Figure 6:**
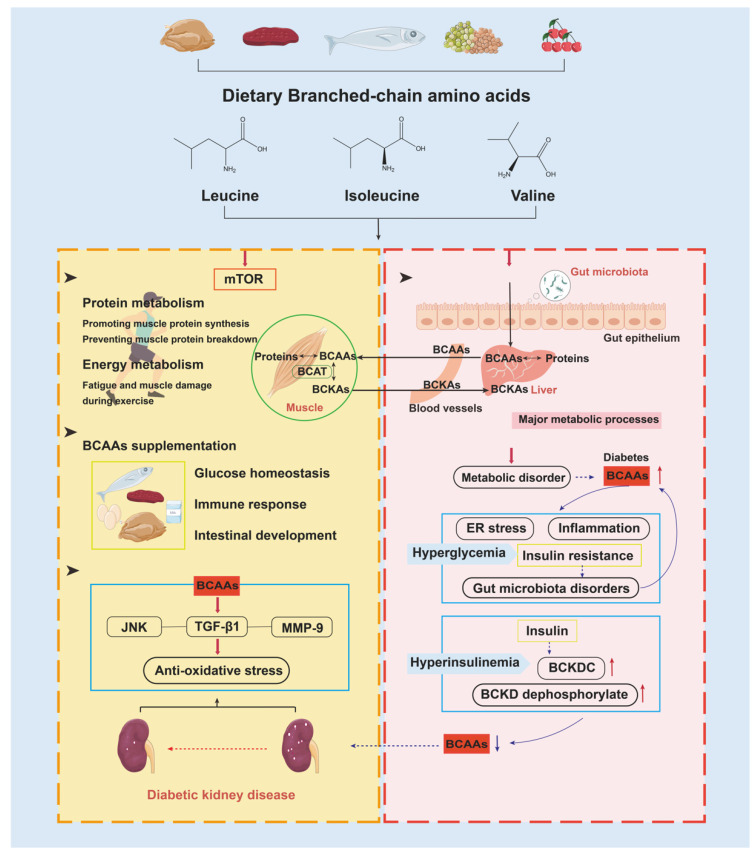
The role of BCAAs and their degradation metabolism in health and disease. The reversible transamination reaction of BCAA catabolism mostly occurs in skeletal muscle. After BCKAs are released back into circulation, most of them are oxidatively decarboxylated in the liver. In health states, BCAA supplementation maintains protein synthesis, energy metabolism, glucose homeostasis, immune response, intestinal development, and renal protection. In diabetic states, excessive BCAA levels, combined with gut microbial dysbiosis, will contribute to hyperglycemia and hyperinsulinemia. Conversely, low BCAA levels will generate renal-function decline. Abbreviations: BCKDC, BCKDC complex; JNK, c-Jun *N*-terminal kinase; MMP-9, matrix metalloproteinase-9.

Branched-chain amino acids (BCAAs) leucine, isoleucine, and valine are all essential AAs that can only be synthesized by bacteria, plants, and fungi, and also can be acquired from dietary chicken breast, beef, tuna, beans, and cherries. In contrast to the majority of AAs, BCAAs are converted to their branched-chain α-keto acids (BCKAs) in skeletal muscle and then released into systemic circulation [72]. BCAAs are well known for their crucial role in promotion of muscle-protein synthesis and modulation of energy metabolism during exercise, which are both mediated through activation of the mTOR signaling pathway [73]. Supplementary BCAAs either act as key nutrition signals or metabolic regulators for glucose homeostasis, immune response, and intestinal development. Also of note is that BCAA supplementation is especially necessary for type 2 diabetes patients with initial renal dysfunction [74]. BCAAs have been reported to counter oxidative stress in the kidneys of diabetic rats and alleviate diabetic kidney injury, such as glomerular hypertrophy, that was mainly mediated via the JNK/TGF-β1/MMP-9 pathway [75] (Figure 6).

In spite of the benefits of BCAAs in metabolic health, studies have also recognized that BCAA homeostasis disruption contributes to pathological conditions of diabetes in animal models of obesity and diabetes [76]. Clinically, strong high-plasma BCAA levels are found in diabetic patients [77]. Under high-glucose circumstances, the capability of muscle protein breakdown is stronger than BCAAs oxidation; combined with serious hypoxia due to inflammation and ER stress, it results in catabolism of BCAAs suppression [73]. Meanwhile, diabetic gut microbial dysbiosis caused by insulin resistance also arrests the degradation metabolism of BCAAs [78,79]. The altered BCAA catabolism predominately results from the altered enzymatic activity of the first two enzymes: branched-chain aminotransferase (BCAT) and branched-chain α-keto acid dehydrogenase (BCKD). In DIO mice, high levels of BCAAs/BCKAs suppressed Akt2 activation and promoted Akt2 ubiquitin–proteasome-dependent degradation through the mTORC2 pathway, depending on the E3 ligase Mul1, finally leading to serious hepatic glucose and lipid metabolic disorder and severe insulin resistance in the liver [80]. As diabetes developed and DKD occurred, plasma BCAA levels began to decline. As long terms of hyperinsulinemia make insulin induce upregulation of the BCKD complex and BCKD dephosphorylation, BCAAs decreased, leading to DKD progression in a 5/6 nephrectomy rat model [81] (Figure 6). Altogether, targeting BCAA metabolism might help prevent not only development of severe glucose and lipid metabolic disorders but also progressive renal dysfunction, especially in patients with diabetes.

## 8. Discussion and Expected Future Prospects

**Figure 7:**
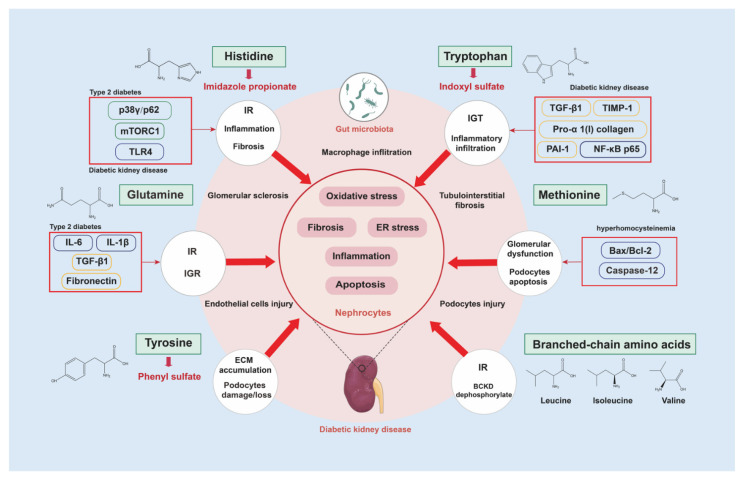
Action mechanisms of altered AA metabolic homeostasis on DKD progression. AAs and their metabolites (i.e., IMP, IS, PS, and Hcy) activate renal oxidative stress, ER stress, inflammation, fibrosis, and the apoptosis relative signaling pathway, which can promote progressive renal damage under diabetic conditions. Abbreviations: IGR, impaired glucose regulation; IGT, impaired glucose tolerance; IR, insulin resistance.

As is known for essential nutrients, AAs are involved in maintenance and regulation of metabolic homeostasis. Under normal circumstances, intake of proper amounts of AAs plays a beneficial role in sustaining functions of the body, such as synthesis of proteins and polypeptide hormones, energy balance, regulation of glucose, and lipid metabolism. This review summarized the benefits of several AAs. In humans, histidine can play an anti-inflammatory, antioxidant, and renal-protective role; tryptophan can regulate glucose metabolism; glutamine can sustain blood glycogen levels; and BCAAs can resist oxidative stress and alleviate renal damage in diabetes. Furthermore, tryptophan showed protective effects on intestinal-barrier injury in vitro, methionine was useful to resistance of oxidative stress in vitro and restoration of fetal kidney dysplasia caused by malnutrition in pregnant rats, and glutamine was reported to inhibit oxidonitrosative stress and protect renal function in STZ-induced rats (Figure 7). 

However, the function of AAs also depends on metabolic homeostasis. Factors such as inappropriate intake, insulin resistance, and intestinal bacteria imbalance will destroy the original metabolic balance of AAs, making them lose their beneficial effects and even causing damage to the body via becoming a pathogenic factor of DKD progression mainly in the basic experimental study stage. For instance, due to diabetic intestinal microbial dysbiosis, abnormal accumulation of intermediate product IMP, derived from histidine, not only damages the intestinal barrier and induces inflammation but also causes insulin resistance, destroys glucose tolerance, and may even be a main factor that leads to DKD. The microbially produced IS derived from tryptophan binds to AHR as a specific ligand and stimulates the NF-κB p65/TGF-β1 signaling pathway to induce renal inflammation and fibrosis. Methionine metabolic disruption not only causes DNA methylation disorder but also leads to abnormal increases of metabolic intermediates Cys and Hcy, causes insulin resistance, induces apoptosis of renal tubular cells and podocytes, and aggravates renal function damage. Excessive glutamine intake will cause renal inflammation and fibrosis via activation of GFPT. Due to intestinal disorder, tyrosine is abnormally converted to PS through the liver metabolism, which can cause thickening of the glomerular basement membrane, destruction of podocytes, vascular inflammation, and fibrosis, leading to proteinuria. Disrupted catabolism of BCAAs blocks the insulin-signaling pathway and causes glucose and lipid-metabolism disorders (Figure 7). This is revisable for either positive or adverse effects of AAs metabolic homeostasis upon occurrence and development of DKD. An imbalance in AA metabolism often results in an increase in harmful circulating metabolites, which leads to alterations in cellular signaling pathways and then directly or indirectly induces DKD progression. Although studies should focus on target clarification, consideration of the connections of metabolic homeostasis of different AAs on the spectrum to explain DKD pathogenesis is more conducive to recovery of AAs metabolism and provides more effective nutrition strategies for clinical treatment of DKD.

In the interaction between AA metabolic homeostasis and DKD, it can be seen that production of many harmful metabolites is greatly affected by disruption of the intestinal barrier and disturbance of the intestinal microbiota [82,83]. Under diabetic conditions, the abundance of intestinal bacteria is significantly reduced, the structure of the intestinal bacteria changes, and the proportion of Bacteroides/Firmicutes phyla is altered [84]. At the same time, a series of omics analyses of intestinal microbial metabolites also showed that many uremic toxins are produced in these conditions. Due to destruction of intestinal microbiota, the immune response increases, and thus the barrier function of the intestinal tract is gradually damaged, resulting in continuous leakage of toxins from the intestinal tract into blood circulation. Nitrogenous groups of AAs are ultimately metabolized and cleared by the kidneys, which means that if the intestinal barrier and the intestinal microbiota are not effectively recovered in the diabetic stage, renal burden will eventually be increased, causing progressive renal dysfunction. Consequently, this paper reviewed the effects of microbially produced toxins, derived from AAs, on DKD development, and also demonstrated potential cellular signaling pathways that they mediate to cause renal injury. Collectively, it is essential for diabetes patients to pay attention to protecting their intestinal barriers and modulate their intestinal microbiota to help reduce intestinal leakage of harmful metabolites at an early stage and better prevent complications of diabetes and DKD from the perspective of nutrition.

The deficiencies of existing diagnostic biomarkers, such as common blood creatinine, urea nitrogen, serum cystatin C, etc., have become increasingly prominent. Clinically, blood creatinine levels have been found to be highly susceptible to internal and external factors such as protein intake and drug and metabolic differences of patients [85]. Blood urea nitrogen is highly susceptible to nephritis [86]. Current shortages have an impact on diagnosis accuracy and lack evidence of nutritional status. According to links between AA homeostasis and DKD progress, some potential biomarkers, such as IMP, IS, Hcy, and PS, mentioned above, appear to be used for clinical diagnosis of DKD. The imbalance in plasma concentration or AA proportion in the body can also be used for disease prediction and diagnosis. For example, valine, Cys, *N*-acetylaspartate, isoleucine, asparagine, betaine, and L-methionine may be the main factors of type 2 diabetes patient progression to DKD, while decreased plasma histidine and valine levels may be used to distinguish DKD patients from type 2 diabetes patients and healthy controls [8,87]. Particularly, it has been clarified that BCAAs and related metabolites are recognized as potential biomarkers of obesity, insulin resistance, type 2 diabetes, and cardiovascular disease in human cohorts [88,89]. Moreover, it has also been confirmed that modulation of BCAA catabolism is helpful in improvement of diabetes progression [90], which is further presented by sodium/glucose cotransporter 2 inhibition [91,92]. Changes in AA metabolic homeostasis in diabetes progression are expected to be applied for prediction and diagnosis of DKD. In fact, integrative analysis of metabolite biomarkers, including valine, leucine, isoleucine, proline, tyrosine, lysine, glutamate, glycine, alanine, palmitic acid, 2-aminoadipic acid serine, and citrulline, has been established for clinical diagnosis and treatment of prediabetes and type 2 diabetes [93,94]. Nowadays, taking advantage of machine learning to analyze large data from genomics, epigenetics, transcriptomics, proteomics, and metabolomics in real-world clinical applications provides a great opportunity to develop multiple biomarkers and avoid information fluctuation of single or isolated biomarkers on overall evaluation efficiency. For clinical prediction and diagnosis of DKD progression, it may be more suitable to carry out a comprehensive evaluation based on the AA homeostasis spectrum and the whole metabolism condition.

Altogether, for patients with nondialysis-dependent CKD, in addition to the advised dietary protein intake of 0.8 g/kg body weight/day recommended by experts [95], the role of AA metabolic homeostasis should be considered comprehensively. For example, it is appropriate for DKD patients to supplement histidine and BCAAs and limit intake of tryptophan, methionine, glutamine, and tyrosine. At the same time, we also need to pay attention to AA proportion, and constantly optimize the protein diet.
